# Relationship of ostial pulmonary vein scar with reduction in pulmonary vein size after atrial fibrillation ablation

**DOI:** 10.1186/1532-429X-11-S1-O73

**Published:** 2009-01-28

**Authors:** Thomas H Hauser, Dana C Peters, John Wylie, Catherine Lau, Mark E Josephson, Warren J Manning

**Affiliations:** Beth Israel Deaconess Medical Center, Boston, MA USA

**Keywords:** Atrial Fibrillation, Cross Sectional Area, Cardiovascular Magnetic Resonance, Pulmonary Vein, Left Atrium

## Introduction

Atrial fibrillation (AF) is the most common sustained arrhythmia. Ablation procedures to electrically isolate the pulmonary veins (PV) from the left atrium have become increasingly popular for the prevention of recurrent AF. PV stenosis is a rare but serious complication of the procedure, thought to be due to scarring of the PV. Previous studies have shown that the intensity of ablation is related to the reduction in PV size after the procedure, but direct assessment of scar in patients has not been performed.

## Purpose

We sought to define the relationship of the change PV size after AF ablation with ostial PV scar as determined by late gadolinium enhancement (LGE) cardiovascular magnetic resonance (CMR).

## Methods

We performed 3D breath-held contrast-enhanced CMR angiography of the PV before and after AF ablation using a 1.5 T MR system. The diameter and cross sectional area (CSA) were determined in the sagittal plane using a previously published method. LGE CMR of the left atrium and PV was obtained after AF ablation using a high-resolution, 3D, navigator gated technique. The scar volume at the ostium of each PV was measured using a threshold technique. The scar volume was normalized to the PV CSA. The change in PV diameter and CSA was expressed as the percentage change compared to the pre-AF ablation PV measurement. The change in PV size before and after evaluation was evaluated using a paired T test. The relationship of the change in PV diameter and CSA to ostial PV scar was evaluated with standard correlation and linear regression.

## Results

**Figure 1:**
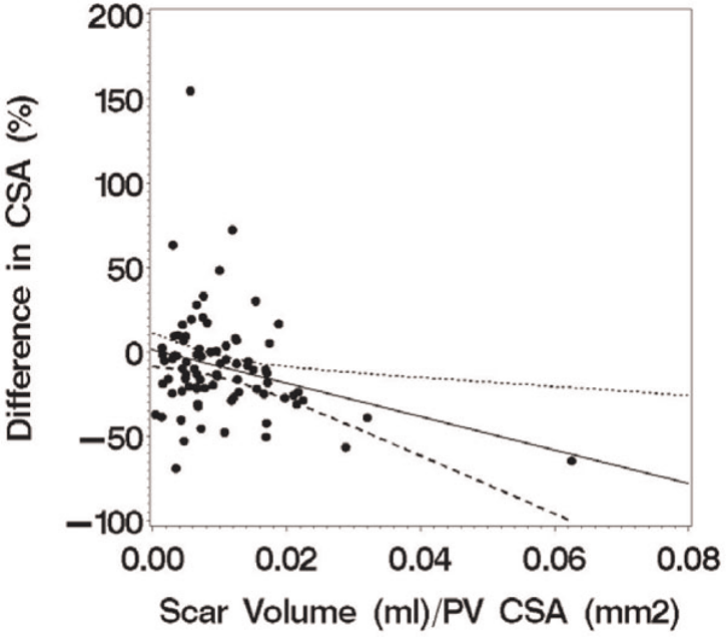
Figure 1

The study cohort was comprised of 23 subjects (3 women, age 58 ± 13 years). CMR was performed 41 ± 17 days after AF ablation. The left sided PV had a common origin in 5 subjects. Scar could not be assessed in one PV due to artifact. A total of 85 PV were available for analysis. Mean PV diameter was 22 ± 7 mm before ablation and declined to 21 ± 6 mm after (p = 0.001) while mean PV CSA declined from 285 ± 141 mm^2^ before ablation to 246 ± 110 mm^2^ after (p < 0.001). A significant correlation was found between ostial PV scar with both the change in PV diameter (r = -0.21, p = 0.049) and was even stronger with PV CSA (r = -0.28, p = 0.010, figure 1).

## Conclusion

PV diameter and CSA significantly decrease after AF ablation. There is a linear relationship between these changes and the magnitude of PV scar as measured by LGE MR.

